# Gallbladder Duplication Associated with Duodenal Atresia

**Published:** 2013-10-01

**Authors:** ML Girish, MM Keshav, BV Raghunath, B Sunil

**Affiliations:** Department of Pediatric Surgery, Rajarajeswari Medical College and Hospital, Bangalore; 1 Department of Pediatrics, Rajarajeswari Medical College and Hospital, Bangalore

**Keywords:** Duodenal atresia, Gall bladder duplication

## Abstract

Gallbladder duplication is an extremely rare anomaly. Association of gall bladder agenesis with duodenal atresia and biliary atresia has been described. However, association of gall bladder duplication with duodenal atresia hasn’t been described so far; we report a case in view of its rarity.

## CASE REPORT

A 3-day-old male neonate, delivered at term by vaginal delivery, weighing 2.75kg, as referred to our institute with antenatal diagnosis of duodenal atresia. Mother was a non-diabetic and had polyhydramnios. Antenatal scan in the second trimester had shown the presence of duodenal atresia; the gall bladder was reported normal. On examination, child was hemodynamically stable with mild icterus. Nasogastric aspirate was dark bilious. X-ray of the abdomen showed classic double bubble sign. After initial resuscitation, the child was taken up for laparotomy. Classic type 3 duodenal atresia was noted. This was associated with a duplicated Boyden type D gall bladder (Fig. 1). No other anomalies were detected. Kimura’s diamond shaped duodeno-duodenostomy was carried out. Since the child was small and there was no problem in biliary drainage, cholecystectomy was not done. The post operative course was uneventful. Child was started oral feeds on post operative day 6 and discharged on post operative day 8. Retrospectively, after reviewing the literature on gall bladder duplications, we felt cholecystectomy should have been done to prevent future complications.

**Figure:**
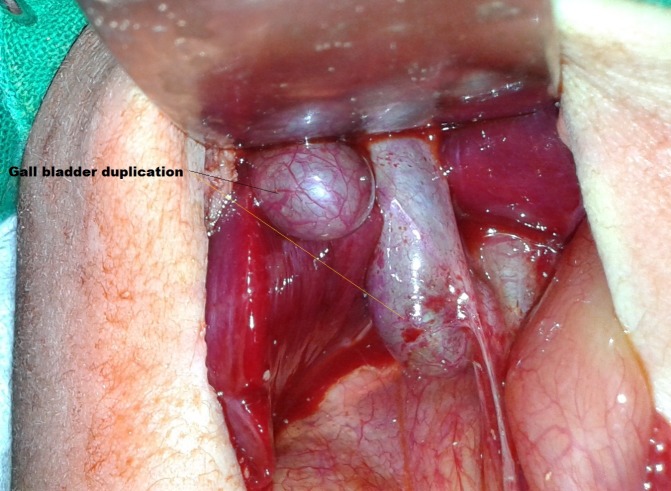
Figure 1: Intra-operative picture showing duplication of gall bladder

## DISCUSSION

Gallbladder duplication is a rare anomaly reported to occur in 1 per 12,000 cholecystograms and 1 per 4000 autopsy specimens.[1] True gall bladder duplication is said to be present when two separate cystic ducts are identified. Boyden classified gall bladder duplications into six types A) Septate gallbladder, B) Fundic duplication, C) Body duplication, D) Y-shaped gallbladder, E) Complete duplication, and F) Bilateral gallbladders.[2]

Though the exact etio-pathogenesis of this condition is unclear, various theories proposed include

1. Incomplete re-vacuolization of the primitive gallbladder, resulting in a persistent longitudinal septum that divides the gall bladder lengthwise
2. Occurrence of two separate cystic buds.

The duplicated cystic ducts may enter the common duct separately or form a Y configuration before a common entrance.[3]

No associated fetal anomalies have been previously described with gallbladder duplication. In our case, the child had associated type 3 duodenal atresia. Whether a common etiology exists for both these anomalies is unclear.

Though, gall bladder duplication can be antenatally identified, it is often mistaken for other common diagnoses like choledochal cyst, folded gall bladder, and gall bladder diverticulum. Detection of this finding is useful because of the high prevalence of cholelithiasis and intermittent cystic duct obstruction that has been reported in at least one of the gall bladders.[4]

Complications associated with double gallbladder include torsion and the development of papilloma, carcinoma, common duct obstruction, and secondary biliary cirrhosis.[5]

Treatment of gall bladder duplication entails removal of both gall bladders. However, in our case, since the neonate was undergoing a major surgery and there was no problem with biliary drainage, cholecystectomy was not contemplated. Retrospectively, after reviewing the literature, we felt cholecystectomy should have been done at the same sitting in order to prevent later complications. However, the infant is on close follow up and doing well. The parents have been explained that the child will be requiring cholecystectomy later.

## Footnotes

**Source of Support:** Nil

**Conflict of Interest:** None

